# Supplementary material for: The role of high-risk geographies in the perpetuation of the HIV epidemic in rural South Africa: A spatial molecular epidemiology study
Source: PLOS Glob Public Health. 2022 Feb 22;2(2):e0000105. doi: 10.1371/journal.pgph.0000105 (PMC10021703; doi:10.1371/journal.pgph.0000105)
Supplement: S1 Text — (PDF) [file pgph.0000105.s001.pdf]

Supplementary Materials for:

The role of high-risk geographies in the perpetuation of the HIV epidemic in rural South Africa: A spatial molecular epidemiology study

Contents

1. Supplementary methods 1
1.1. Rationale 1
1.2. Data sources 3
2.1. Simulation models 5
2.1.1. Model 1. Random link formation model 6
2.1.2. Model 2. Gravity model 6
2.1.3. Model fitting 7
2.1.4. Goodness of fit of the models 8
3. Supplementary results 8
3.1. Intra-household transmission 8
3.2. Phylogenetic cluster 9
3.3. Microsimulation results 10
3.4. Spatial variables 11
3. References 13

1. Supplementary methods

1.1. Rationale

The structure of human contact networks both facilitates and constrains the spread of pathogens. Particularly for HIV infection, the epidemic emerges first in specific subpopulations whose behavioural characteristics expose them to a higher risk of HIV infection [1]. In most countries the HIV epidemics remain concentrated in these high-risk subpopulations (core groups) including men who have sex with men (MSM), injecting drug users (IDUs), and female sex workers (FSWs) [2]. The high connectivity between individuals that are part of these subpopulations boost the transmission, maintaining a sustainable HIV epidemic (basic reproductive number [$R_0 > 1$]). In concentrated epidemics several factors, including the configuration of the network, prevent the dispersion of the epidemic into the general population

(Figure S1 A). Besides few outflows of infections from the core groups, the epidemic remains enclosed in the core groups and is not sustainable in the general low-risk population ($R_0 < 1$).

The realization that heterogeneity in contact numbers can have a large impact on the transmission of a pathogen has produced considerable interest in networks in epidemiology. Through the study of network models, it would be possible to understand that contact network structure has a profound influence on epidemic dynamics and whether control strategies will be effective [3, 4]. Yet data on human contact networks are notoriously difficult to gather. Novel surveillance methodologies have been developed to study HIV epidemiology among high-risk subpopulations [2, 5-8]. Repeated integrated bio-behavioral surveillance surveys that incorporate state of the art sampling methodologies to reach hidden populations, such as respondent driven sampling, network scale-up, and capture-recapture methods, provide the best approach to study these key core groups [9-11]. Likewise, control interventions have been designed to defeat the epidemic in these core groups, and countries are moving towards allocative efficiency resource allocation targeting these high-risk subpopulations (Figure S1 B).

The HIV epidemic in several countries in sub-Saharan Africa (SSA), however, has escaped from the high-risk subpopulations (Figure S1 C), and successfully invaded and maintained sustainable levels of transmission in the general population ($R_0 > 1$). This complex epidemiological context challenges the concept of targeted interventions to behavioral core groups, and difficulties the identification of vulnerable population at high risk of infection.

Recent evidence suggests that the HIV epidemic in SSA is characterized by a spatially structured transmission of the infection, with geographically located micro-epidemics (geographical HIV clusters) [12-15]. In this study, we argue that these geographical HIV clusters expose the location of vulnerable population at high risk of infection. Likewise, new hope for the empirical study of contact networks has emerged in recent years from the widespread availability of pathogen molecular sequence data. Phylogenetic analysis of HIV sequences is an excellent adjunct to enumerating networks and allows for tracking of local transmission patterns. In epidemiological phylogenetic analysis, sequence data is already commonly used to link individuals into probable transmission pairs or clusters based on the evolutionary relationship between their pathogens. While such approaches cannot fully reveal the structure of contact networks due to the inherent limitations of sampling, phylogenetic approaches to study pathogen spread provide a useful heuristic for identifying networks and assessing the connectivity between different subpopulations or risk-groups.

Against this background, here for the first time a spatially explicit phylogenetic transmission network of a community with a generalized HIV epidemic and the transmission intensity from an HIV high-risk location were analyzed. We argue that if the HIV geographical high-risk populations have a role similar to the one played by behavioral core groups in transmission networks of concentrated epidemics then they could be key drivers of the transmission dynamics of the generalized epidemic. Targeting these geographical core groups, would not only impact

HIV incidence within the geographical HIV cluster, but also disrupt the entire transmission network of the community (Figure S1 D).

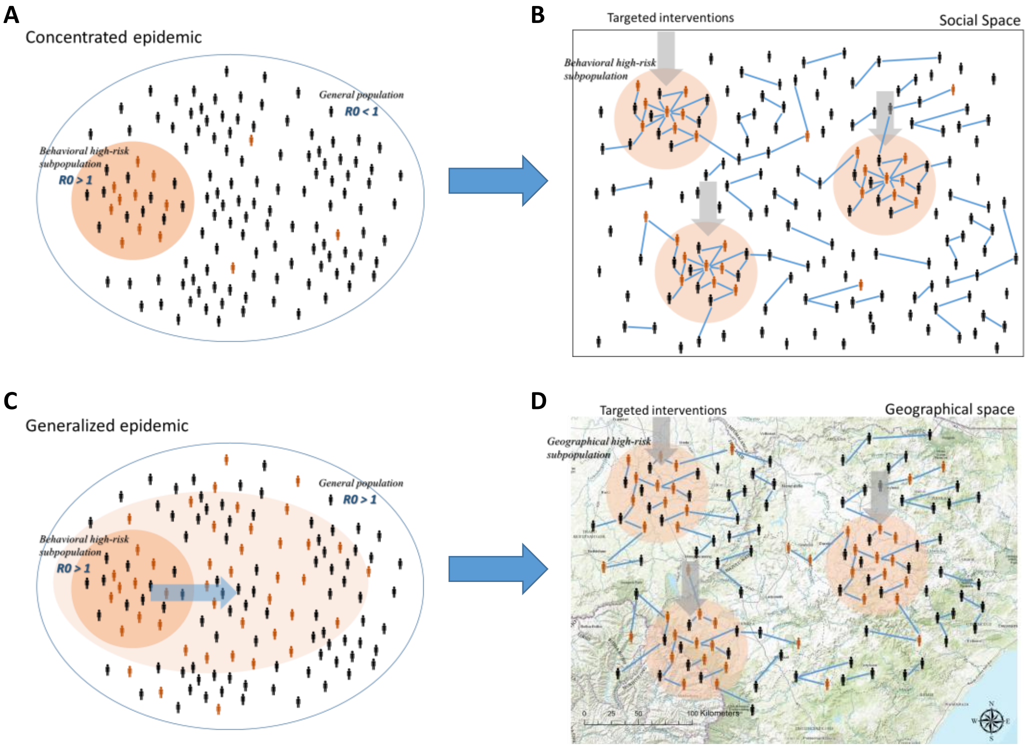

Figure S1. **A)** In most countries the HIV epidemics remain concentrated in these high-risk subpopulations (core groups. **B)** Control interventions target behavioral core groups in concentrated epidemics. **C)** HIV epidemic invades the general population in generalized epidemics. **D)** Control interventions target geographical core groups in generalized epidemics. Black figures represent susceptible individuals, whereas red figures represent infected individuals. Maps were created using ArcGIS by Esri version 10.5 (http://www.esri.com) [16], and basemaps were obtained from ESRI and OpenStreetMap available at ArcGIS Online basemaps (https://www.arcgis.com/home/item.html?id=b834a68d7a484c5fb473d4ba90d35e71).

1.2. Data sources

2. Data come from one of the most comprehensive demographic surveillance systems in Africa— the Africa Health Research Institute (AHRI) Population Intervention Platform Surveillance Area (PIPSA)[17]. This surveillance system is located in Hlabisa subdistrict, one of the five subdistricts in the rural district of Umkhanyakude in northern KwaZulu-Natal, South Africa (Figure S2). This surveillance system has routinely collected socio-demographic, behavioral and epidemiological information on a population of ~90,000 participants within a circumscribed geographic area (438 km2) for over a decade (Figure S2).

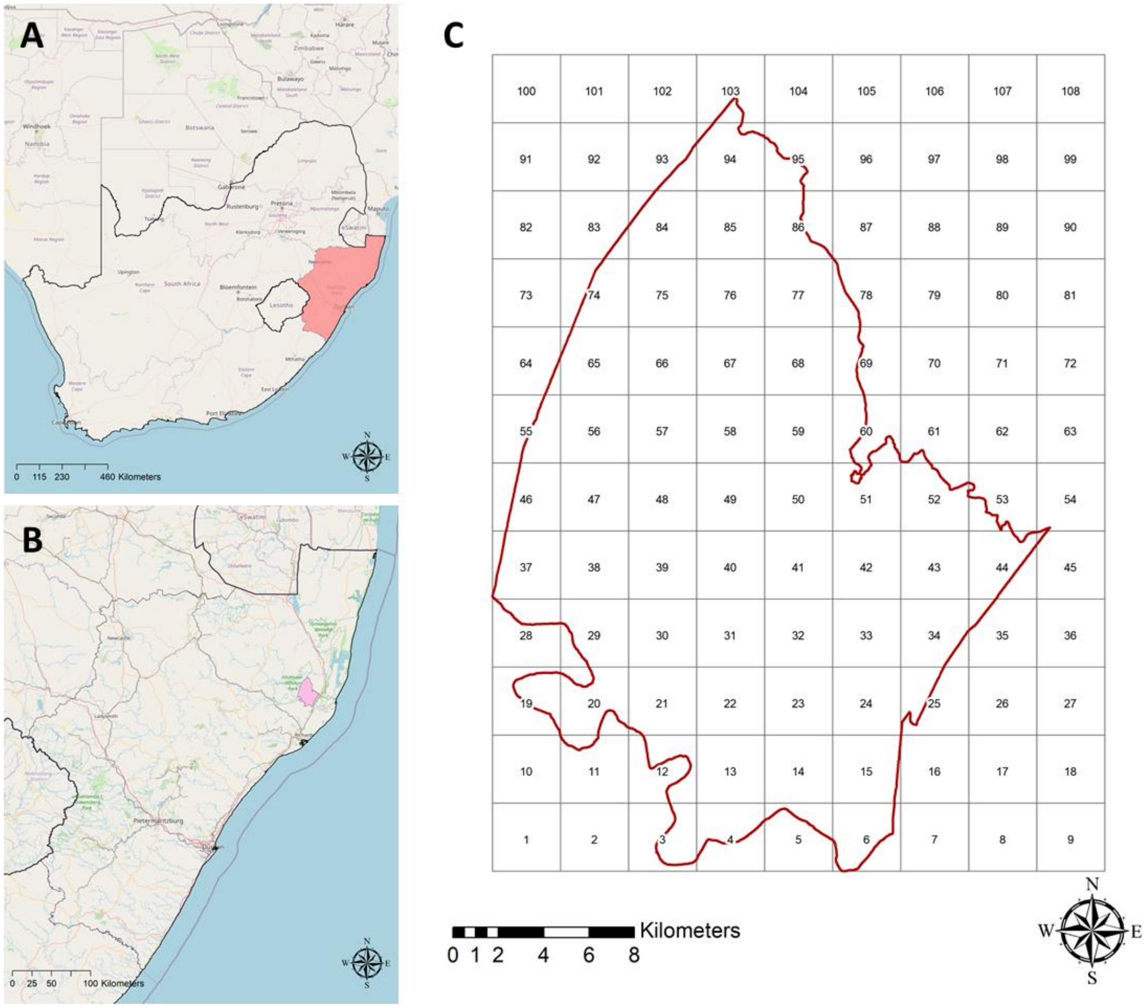

Figure S2. **A)** location of the Kwazulu-Natal province (red) in South Africa; **B)** a close-up of the Kwazulu-Natal province with the area sampled (red); **C)** grid composed by 108 cells of 3km X 3km dimension that covered the entire surveillance area for spatial aggregation of the data. Maps were created using ArcGIS by Esri version 10.5 (http://www.esri.com) [16], and basemaps were obtained from ESRI and OpenStreetMap available at ArcGIS Online basemaps (https://www.arcgis.com/home/item.html?id=b834a68d7a484c5fb473d4ba90d35e71).

Between Jan 1, 2011 and Dec 31, 2014, 36,035 individuals between 15-54 registered in the surveillance were residents in the study area. Of these, 26,984 individuals were offered to participate in the population-based annual HIV testing. Of these, 18,548 individuals (for a total of 29,030 records) consented and participated in the annual HIV testing. All of them consented and provided dried blood spots samples. Of these 5, 619 individuals were HIV-positive, and 1,426 samples with >10,000 RNA copies/ml were included for phylogenetic analyses (Figure S3).

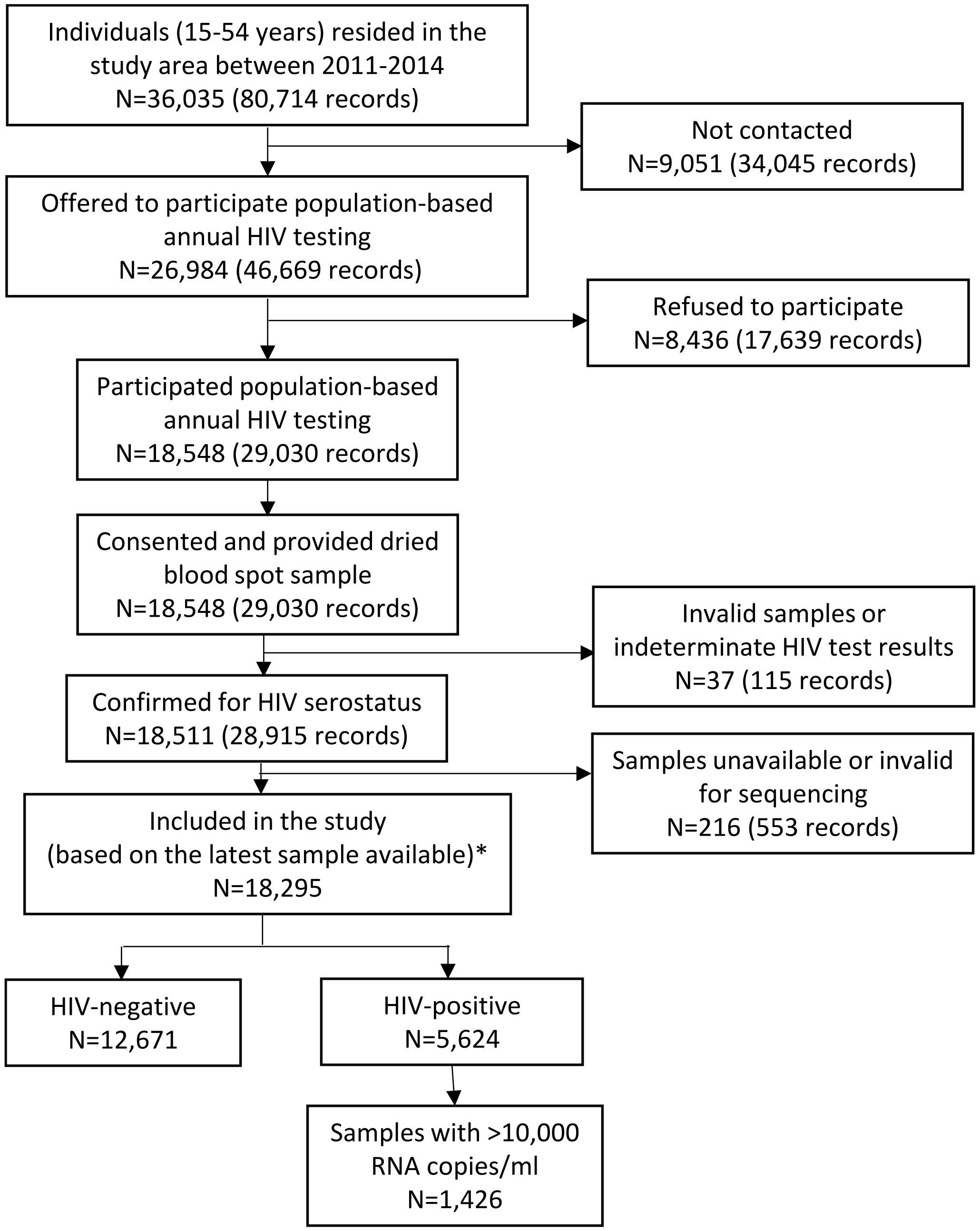

Figure S3. Flow chart of study population

2.1. Simulation models

Two microsimulation models were designed to assess the association between the HIV geographical high-risk population and the transmission link configuration observed. All two microsimulations used data from the total sampled population, in which the geo-location of each participant as well as the number of transmission links were used. In each microsimulation, the number of links subject to the location of participants with respect to the HIV geographical high-

risk populations (within, outside, or in both sides of the geographical HIV cluster) as well as the distance of the links were recorded. All results were based on the average of 10,000 realizations of the model.

2.1.1. Model 1. Random link formation model

For this model, the probability of a link formation between individuals is independent of the distance between individuals or the location of the individuals related to the geographical HIV cluster. The algorithm randomly selects two individuals and generates a link between them. The microsimulation repeats this process until the number of links (n = 350) is reached. The flow diagram in Figure S4 illustrates the steps of this model.

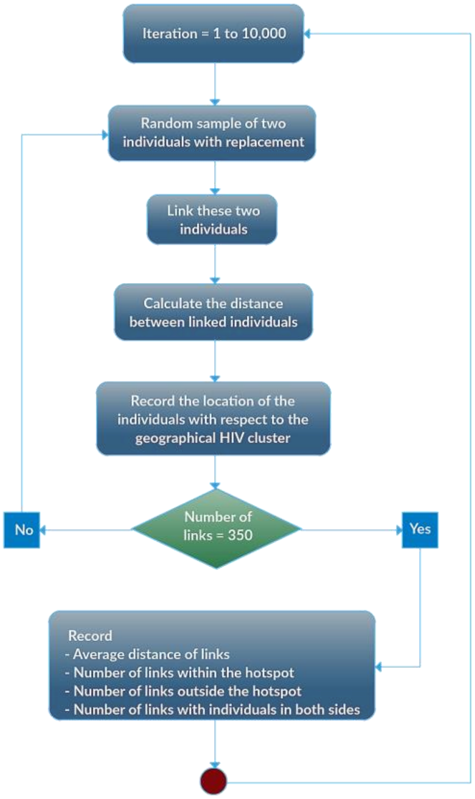

Figure S4. Flow diagram for the random link formation model

2.1.2. Model 2. Gravity model

For the gravity model, in the first step two individuals are selected based on their location with respect to the geographical HIV cluster, in which individuals located within the geographical HIV cluster have 40% higher likelihood (value derived from the HIV spatial risk estimation), of being selected for a link formation. Then, the distance between individuals is measured, and the location of the individuals related to the geographical HIV cluster (if the individuals are located within or outside the geographical HIV cluster) is recorded. For this microsimulation, in the first step an individual is randomly selected. In the next step, the distance between individuals is

measured and the probability of link formation is estimated using the following exponential decay function,

$$Probability\ of\ link\ \ formation = e^{-\lambda * dist} \qquad (1)$$

In which λ is a fitted parameter, and *dist* is the distance between individuals being evaluated, and if one of the individuals is located within the geographical HIV cluster, the likelihood of ling formation is increased by 40%. The flow diagram in Figure S5 illustrates the steps of this model.

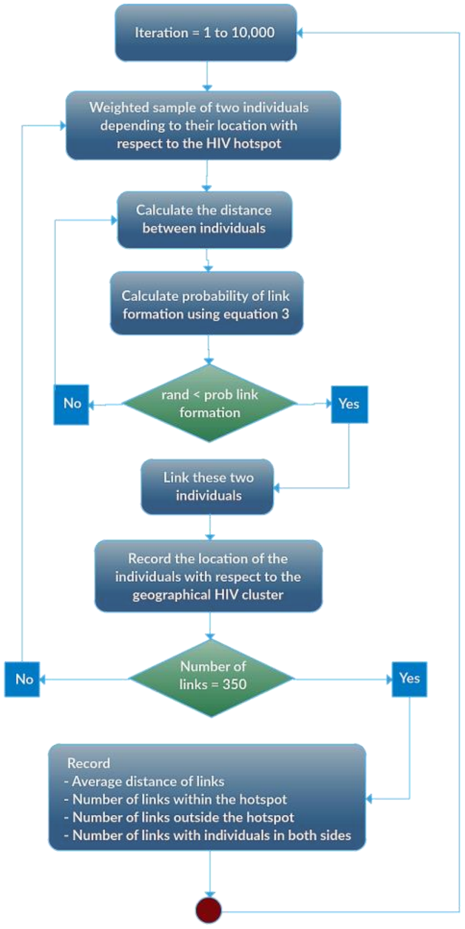

Figure S5. Flow diagram for the gravity model

2.1.3. Model fitting

Model 2 was fitted to six measures derived from the transmission link data: number of links within, outside and in both sides of the geographical HIV cluster, and average distance of links within, outside and in both sides of the geographical core area. Fitting procedure was performed using a nonlinear least-square fitting method that incorporates an algorithm implemented in

MATLAB® [18] that minimizes the sum of squares between all measures and the model, using the Nelder–Mead simplex algorithm as described elsewhere [19].

2.1.4. Goodness of fit of the models

The evaluation of the performance of the two different microsimulations was conducted using the root mean squared error (RMSE) approach. After conducting 10,000 realizations of the model, the RMSE of each of the six measures fitted were calculated. Each RMSE was calculated as the square root of the average squared difference between the observed measure *i* (O_i) and the measure estimated by the model (E_i) as following,

$$RMSE = \sqrt{\frac{\sum_{i=1}^{N}\left(E_i - O_i\right)^2}{N}} \qquad (2)$$

3. Supplementary results

3.1. Intra-household transmission

Figure S6 illustrates the location of heterosexual links located in the same household (red dots), and the location of participants involved in a heterosexual link that were located in different households (green dots). The map only included participants from heterosexual links in which age difference between the couples was ≤5 years.

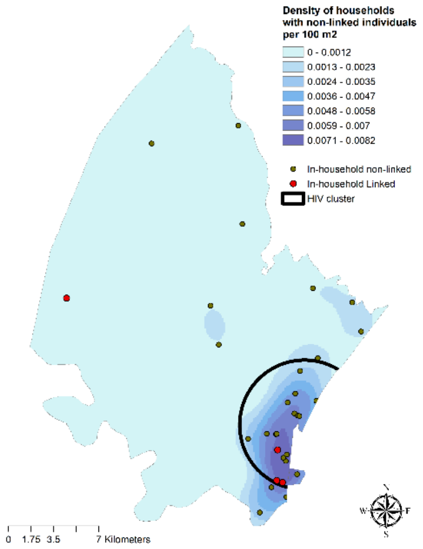

Figure S6. Geographical location (green dots) and kernel density map of households in which a male and a female living in the same house were not phylogenetically linked (n= 29), and households in which a male and a female living in the same house were phylogenetically linked (n= 4) (red dots). Black circle enclosed the geographical HIV cluster. Maps were created using ArcGIS by Esri version 10.5 (http://www.esri.com) [16].

3.2. Phylogenetic cluster

From the 1,426 HIV Pol sequences included in the phylogenetic analyzes, the homestead geo-location of the sampled individual was available for 1,222 sequences. From these 1,222 individuals we identified that 333 were linked in 132 phylogenetic transmission clusters with sizes ranging from 2 sequences to 11 (Figure S7 A). These clustered individuals accounted for a total of 350 transmission links, whose geo-location is illustrated in Figure S8 A. The geo-location of non-phylogenetically linked individuals is illustrated in Figure S8 B. The proportion of individuals sampled from inside the identified geographical HIV cluster was roughly the same among individuals in phylogenetic cluster and in the whole dataset of sequences (Figure S7 B)

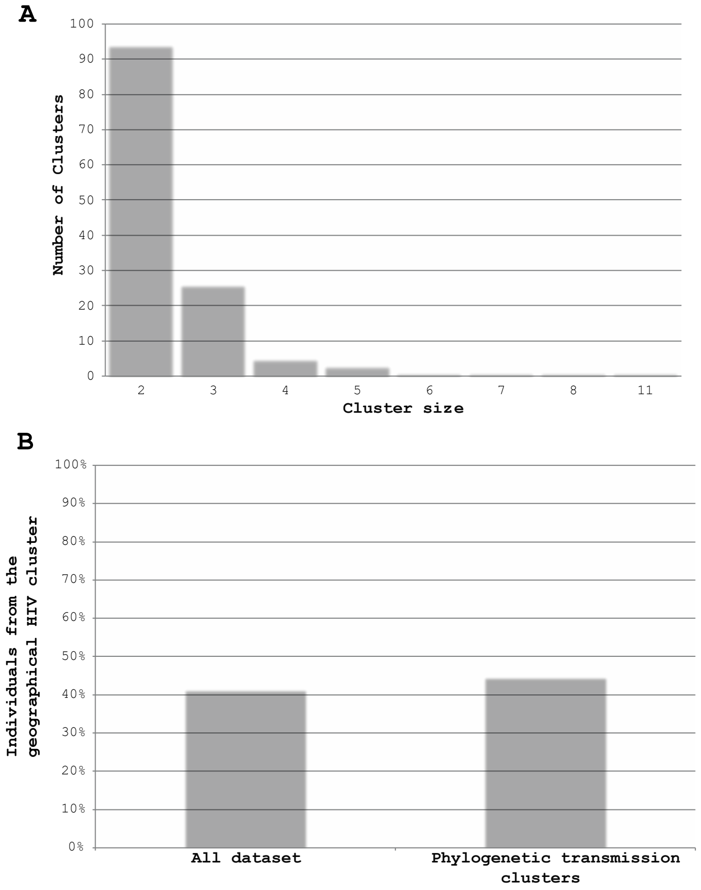

Figure S7. A) Cluster sizes distribution for the identified phylogenetic transmission clusters. **B)** Proportion of samples from the geographic HIV cluster in each analyzed group of sequences.

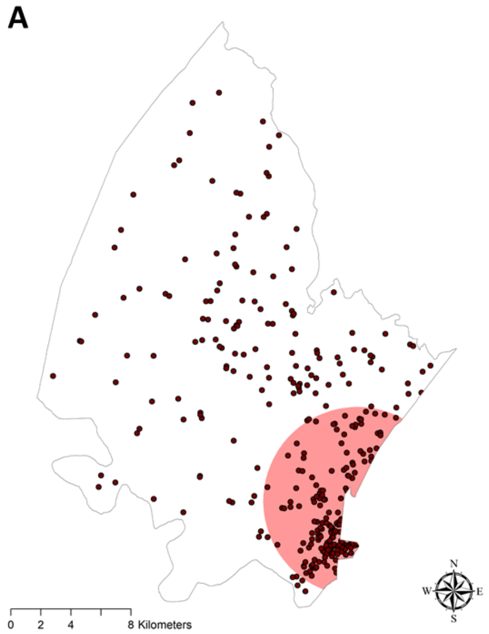

Figure S8. A) Geographical distribution of participants that were phylogenetically linked in a transmission cluster, and **B)** participants that were not phylogenetically linked. Red circle indicates the location of the geographical HIV cluster. Spatial random error in the geographical references was introduced. Maps were created using ArcGIS by Esri version 10.5 (http://www.esri.com) [16].

3.3. Microsimulation results

Figure S9 illustrates the results of one of the realizations of each model compared with the transmission link data.

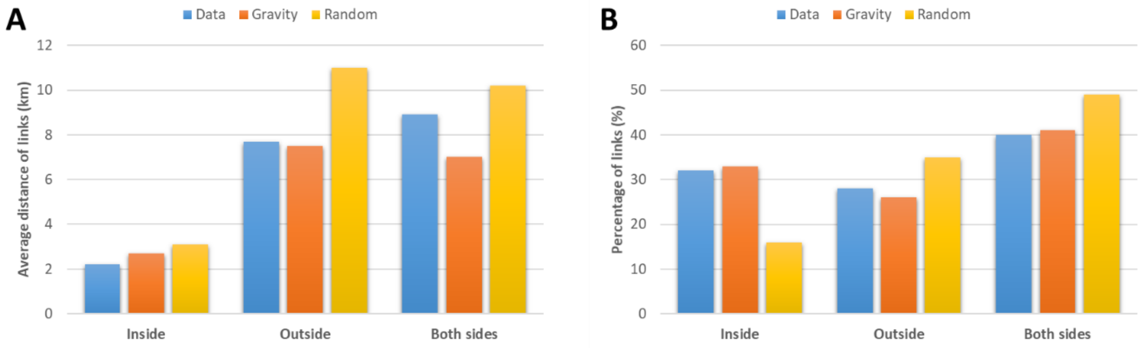

Figure S9. Comparisons among average distance of transmission links derived from the models **(A)** and distribution of transmission links **(B).** Distribution of the transmission links derived from the random model (yellow bars; RMSE = 113.17). Distribution of the transmission links derived from the gravity model (orange bards; RMSE = 16.37).

3.4. Spatial variables

The geographical HIV cluster identified is located in a high population density area and high urban development (Figure S10). Individuals outside to the geographical core area and that belong to an HIV transmission link appears to be dispersed across the study area without a clear spatial pattern (Figure S11).

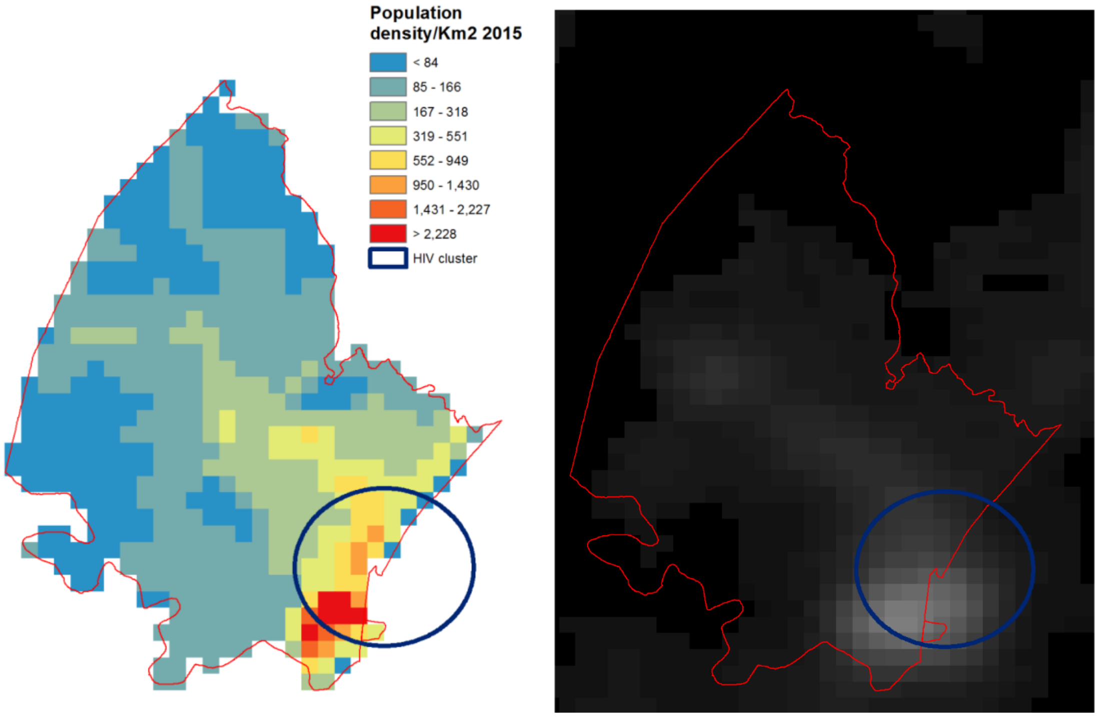

Figure S10. Population density [20] and nightlight intensity [21] in the area of study. Blue circle enclosed the geographical HIV cluster. Maps were created using ArcGIS by Esri version 10.5 (http://www.esri.com) [16].

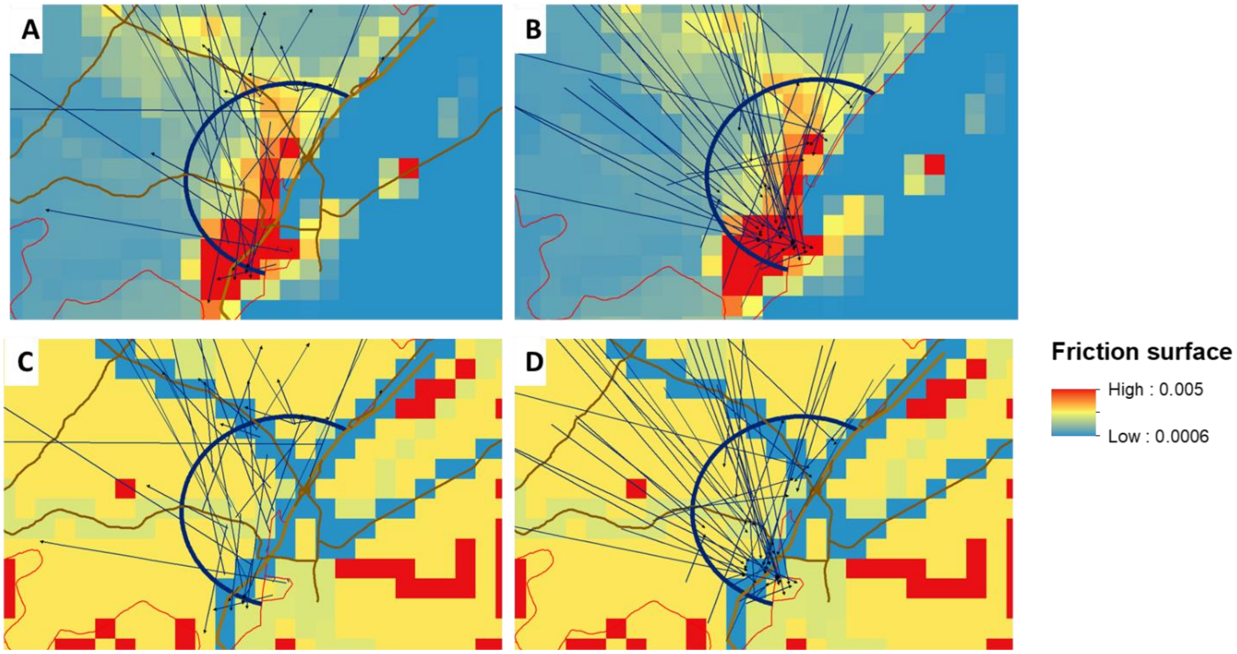

Figure S11. Transmission link locations with respect to population density within the geographical HIV cluster [20] (**A, B).** Transmission link locations with respect to accessibility [22] within the geographical HIV cluster **(C, D).** Blue circles enclosed the geographical HIV cluster. Spatial random error in the geographical references was introduced. Maps were created using ArcGIS by Esri version 10.5 (http://www.esri.com) [16].

3. References

1. Anderson R, May R. Infectious Diseases of Humans: Dynamics And Control. Illustrated r, editor: Oxford University Press; 1991. 757 p.
2. Mills S, Saidel T, Magnani R, Brown T. Surveillance and modelling of HIV, STI, and risk behaviours in concentrated HIV epidemics. Sexually transmitted infections. 2004;80(suppl 2):ii57-ii62.
3. Meyers LA, Pourbohloul B, Newman ME, Skowronski DM, Brunham RC. Network theory and SARS: predicting outbreak diversity. Journal of theoretical biology. 2005;232(1):71-81.
4. Bansal S, Grenfell BT, Meyers LA. When individual behaviour matters: homogeneous and network models in epidemiology. Journal of the Royal Society Interface. 2007;4(16):879-91.
5. Rehle T, Lazzari S, Dallabetta G, Asamoah-Odei E. Second-generation HIV surveillance: better data for decision-making. Bulletin of the World Health Organization. 2004;82:121-7.
6. Diaz T, Garcia-Calleja JM, Ghys PD, Sabin K. Advances and future directions in HIV surveillance in low- and middle-income countries. Current Opinion in HIV and AIDS. 2009;4(4):253-9 10.1097/COH.0b013e32832c1898.
7. Heckathorn DD. Respondent-Driven Sampling: A New Approach to the Study of Hidden Populations. Social Problems. 1997;44(2):174-99. doi: 10.2307/3096941.
8. Berchenko Y, Frost SDW. Capture-recapture methods and respondent-driven sampling: their potential and limitations. Sexually Transmitted Infections. 2011;87(4):267-8. doi: 10.1136/sti.2011.049171.
9. Reintjes R, Wiessing L. 2nd-generation HIV surveillance and injecting drug use: uncovering the epidemiological ice-berg. Int J Public Health. 2007;52(3):166-72. doi: 10.1007/s00038-007-5123-0.
10. Ezoe S, Morooka T, Noda T, Sabin ML, Koike S. Population Size Estimation of Men Who Have Sex with Men through the Network Scale-Up Method in Japan. PLoS ONE. 2012;7(1):e31184. doi: 10.1371/journal.pone.0031184.
11. Bloor M, Leyland A, Barnard M, McKeganey N. Estimating hidden populations: a new method of calculating the prevalence of drug-injecting and non-injecting female street prostitution. British Journal of Addiction. 1991;86(11):1477-83. doi: 10.1111/j.1360-0443.1991.tb01733.x.
12. Cuadros DF, Awad SF, Abu-Raddad LJ. Mapping HIV clustering: a strategy for identifying populations at high risk ofHIV infection in sub-Saharan Africa. International Journal of Health Geographics. 2013;12(1):28. doi: 10.1186/1476-072x-12-28.
13. Fichtenberg CM, Ellen JM. Moving from core groups to risk spaces. Sexually transmitted diseases. 2003;30(11):825-6.
14. Hallett T, Anderson S-J, Asante CA, Bartlett N, Bendaud V, Bhatt S, et al. Evaluation of geospatial methods to generate subnational HIV prevalence estimates for local level planning. AIDS. 2016;30(9):1467-74.
15. Aral SO, Torrone E, Bernstein K. Geographical targeting to improve progression through the sexually transmitted infection/HIV treatment continua in different populations. Current Opinion in HIV and AIDS. 2015;10(6):477-82.
16. ESRI. ArcGIS 10.x. Redlands, CA, USA: ESRI. 2004.

17. Bärnighausen T, Tanser F, Gqwede Z, Mbizana C, Herbst K, Newell ML. High HIV incidence in a community with high HIV prevalence in rural South Africa: findings from a prospective population-based study. AIDS. 2008;22(1):139-44. PubMed PMID: 18090402.
18. MATLAB. MATLAB 2014a, The MathWorks, Natick, 2014.
19. Lagarias JC, Reeds JA, Wright MH, Wright PE. Convergence properties of the Nelder--Mead simplex method in low dimensions. SIAM Journal on optimization. 1998;9(1):112-47.
20. Tatem AJ. WorldPop, open data for spatial demography. Scientific data. 2017;4.
21. Mellander C, Lobo J, Stolarick K, Matheson Z. Night-time light data: A good proxy measure for economic activity? PloS one. 2015;10(10):e0139779.
22. Weiss D, Nelson A, Gibson H, Temperley W, Peedell S, Lieber A, et al. A global map of travel time to cities to assess inequalities in accessibility in 2015. Nature. 2018;553(7688):333.
