# Supplementary material for: The role of high-risk geographies in the perpetuation of the HIV epidemic in rural South Africa: A spatial molecular epidemiology study
Source: PLOS Glob Public Health. 2022 Feb 22;2(2):e0000105. doi: 10.1371/journal.pgph.0000105 (PMC10021703; doi:10.1371/journal.pgph.0000105)
Supplement: Attachment — Submitted filename: Reviewer_Comments_102421.pdf [file pgph.0000105.s002.pdf]

The role of high-risk geographies in the perpetuation of the HIV epidemic in rural South Africa

Diego F. Cuadros, Tulio de Oliveira, Tiago Gräf, Dennis M. Junqueira, Eduan Wilkinson, Philippe Lemey, Till Bärnighausen, Hae-Young Kim, Frank Tanser

RESPONSE TO REVIEWERS' COMMENTS

We appreciate the opportunity to review our work to the journal. We would like to thank the reviewers and the editor for assessing our work and for their valuable feedback and suggestions that have improved our manuscript. Please find below a point-by-point reply that addresses each of the reviewers' comments. We have also incorporated these suggestions in the revised manuscript as noted below.

Journal Requirements:

1. *We note that participants provided oral consent. Please state in the Methods:*
   - Why written consent could not be obtained
   - Whether the Institutional Review Board (IRB) approved use of oral consent
   - How oral consent was documented
   For more information, please see our guidelines for human subjects research:
   https://journals.plos.org/plosone/s/submission-guidelines#loc-human-subjects-research

- In the Ethic statement we indicated that participants provided written informed consent and not oral consent.

2. Please provide a detailed Financial Disclosure statement. This is published with the article, therefore should be completed in full sentences and contain the exact wording you wish to be published.
i). Please include all sources of funding (financial or material support) for your study. List the grants (with grant number) or organizations (with url) that supported your study, including funding received from your institution.

- Done (lines 442-450 in the marked document)

ii). State the initials, alongside each funding source, of each author to receive each grant.

- Done (lines 443 in the marked document)

iii). State what role the funders took in the study. If the funders had no role in your study, please state: "The funders had no role in study design, data collection and analysis, decision to publish, or preparation of the manuscript."

- Done (lines 448-450 in the marked document)

iv). If any authors received a salary from any of your funders, please state which authors and which funders. If you did not receive any funding for this study, please simply state: "The authors received no specific funding for this work."

- Done

3. Since your data is not available for proprietary reasons, please explain via email why the data is not available. Please also include the contact information for the third party organization that should be contacted should other researchers want to request access to this data and please include the full citation of where the data can be found. We also request that you verify with us via email that any researcher will be able to obtain the data set in the same manner that the you have obtained it. If you feel you are unwilling or unable to adhere to this policy, please explain your reasons by return email and your exemption request will be escalated to the editor for approval. Your exemption request will be handled independently and will not hold up the peer review process, but will need to be resolved should your manuscript be accepted for publication. One of the Editorial team will be in touch if they require more information.

- All data used for this study have been stored In the AHRI data repository (https://data.ahri.org/index.php/home). We have included this link and the contact information for the Research Data Management leader at AHRI in the revised version of the manuscript (lines 210-219 in the marked document).

4. Please provide separate figure files in .tif or .eps format only, and remove any figures embedded in your manuscript file. If you are using LaTeX, you do not need to remove embedded figures.
For more information about figure files please see our guidelines:
https://journals.plos.org/globalpublichealth/s/figures

- Done

5. Please provide us with a direct link to the base layer of the map used in Figures 2, 5, Figures S1, S2, S7, S9, S10, S11, S16, S17 and ensure this location is also included in the figure legend. Please note that, because all PLOS articles are published under a CC BY license (creativecommons.org/licenses/by/4.0/), we cannot publish proprietary maps such as Google Maps, Mapquest or other copyrighted maps. If your map was obtained from a copyrighted source please amend the figure so that the base map used is from an openly available source.
Please note that only the following CC BY licences are compatible with PLOS licence: CC BY 4.0, CC BY 2.0 and CC BY 3.0, meanwhile such licences as CC BY-ND 3.0 and others are not compatible due to additional restrictions. If you are unsure whether you can use a map or not, please do reach out and we will be able to help you.
The following websites are good examples of where you can source open access or public domain maps:
** U.S. Geological Survey (USGS) - All maps are in the public domain. (http://www.usgs.gov)*
** PlaniGlobe - All maps are published under a Creative Commons license so please cite "PlaniGlobe, http://www.planiglobe.com, CC BY 2.0" in the image credit after the caption. (http://www.planiglobe.com/?lang=enl)*

** Natural Earth - All maps are public domain. (http://www.naturalearthdata.com/about/terms-of-use/)*

- -Figures 2 and 5 do not include base layers
  -Figure S1. Link for the OpenStreetMap base layer was included
  -Figure S2. Link for the OpenStreetMap base layer was included
  -Figure S7. Base layer for this map was removed
  -Figure S9. Base layer for these maps were removed
  -Figure S10. These maps were removed from the updated version of the supplementary materials
  -Figure S11. These maps were removed from the updated version of the supplementary materials
  -Figure S16. These maps do not include base layers
  -Figure S17. These maps were removed from the updated version of the supplementary materials

6. Please review your reference list to ensure that it is complete and correct. If you have cited papers that have been retracted, please include the rationale for doing so in the manuscript text, or remove these references and replace them with relevant current references. Any changes to the reference list should be mentioned in the rebuttal letter that accompanies your revised manuscript. If you need to cite a retracted article, indicate the article's retracted status in the References list and also include a citation and full reference for the retraction notice.

- Reference list was reviewed and corrected.

Additional Editor Comments (if provided):

I concur with the reviewers that this is a clearly written manuscript of a study using multiple robust methods to investigate an important public health problem. However, as one of the reviewers noted, the data availability statement does not appear to meet the requirements of the journal: https://journals.plos.org/globalpublichealth/s/data-availability. I realize that this study is based on highly sensitive HIV data. However, please note that a non-author institutional contact at AHRI should be specified in the manuscript: "When possible, we recommend authors deposit restricted data to a repository that allows for controlled data access. If this is not possible, directing data requests to a non-author institutional point of contact, such as a data access or ethics committee, helps guarantee long term stability and availability of data. Providing interested researchers with a durable point of contact ensures data will be accessible even if an author changes email addresses, institutions, or becomes unavailable to answer requests."

- All data used for this study have been stored In the AHRI data repository (https://data.ahri.org/index.php/home). We have included this link and the contact information for the Research Data Management leader at AHRI in the revised version of the manuscript (lines 208-219 in the marked document).

Reviewer #1:

Authors present analysis and identification of high-risk HIV geographical clusters and their role in HIV-1 transmission networks. They make recommendations that identifying high risk geographic spots could aid in intervention programming especially in Sub-Saharan Africa where the epidemic seems to be driven by geographical sources and sinks. In their attempt to answer the Questions "what is the contribution of HIV high-risk locations in the overall HIV transmission network" they we applied spatial epidemiology and phylodynamic analysis to transmission network analysis one of the largest population-based prospective cohorts located in the rural KwaZulu-Natal province of South Africa. The authors demonstrated that spatial and transmission network analysis can identify specific geographical a HIV hyper-endemic epidemic. A large proportion the HIV transmission links identified by phylogenetic analysis were directly linked to the HIV high-risk location. This work is critical for targeting interventions for optimal use of scarce resources.

- We thank the reviewer for the critical assessment of our work and for the positive feedback provide. Please find below a point-by-point reply of each of the comments of the reviewer

Comments

1. Although the authors clearly explain how the attempted to protect the confidentiality of the participants by introducing a geographical random error to the geographical coordinates of each homestead included in the study, the longitudinal cohort is well known and well established. What additional measures or community engagement were used to avoid social injury or stigma attached to the community if the results of this work can be translated back for intervention programming as suggested by the authors for "microtargeted" interventions?

- We thank the reviewer for the thoughtful comment. We agree with the reviewer about the high importance of protecting the communities and avoid any potential social injury. We made substantial efforts to protect confidentiality and avoid any potential stigma generated by our results. Maps included in the study were generated for illustrative purposes only and do not contain any geographical reference (no background maps were included) of the participants or the areas highlighted in our study. Regarding the concern raised by the reviewer, this social assessment of geographically targeted interventions is beyond the main objectives of our study. However, we believe this would be an important component of microtargeted intervention approaches that need to be assessed in further studies. We have now mentioned the importance of the social component of the approach proposed in this study and the need to design and implement studies assessing these social implications (lines 371-375 in the marked document).

2. What was the median between the date of the last negative and first positive HIV test for the incidence estimation cohort?

- The median interval of time between last HIV-negative and first HIV-positive test is 2.18 years. This estimation was reported in lines 92-93 in the marked document.

-

3. What is the proportion of participants with VL <10,1000 not sequenced? What is the potential impact for missing sequences due to lower viral loads (<10, 000 copies/mL) or sequencing prioritisation for higher viral loads on the identification of geographical clusters. Could authors provide analysis of possible impact of these values. Viral loads as low as 2000 copies/mL have associated with onward transmission.

- We want to highlight that sequencing protocols and selection were based on ensuring successful sequencing of the virus and not for virus transmission potential. In addition, the association of high viral loads and a strict method for identify transmission clusters based on genetic distance and branch support increases the confidence on the transmission links identified here and helps reconstruct the recent history of HIV transmission within the community. For that reason, higher viral loads were selected to ensure successful sequencing of the viruses under limited resources availability. Flow diagram in Figure S3 in Supplementary Materials included a detailed description of the final sampled size in which samples from 1,426 HIV+ individuals (25.4%) of the total HIV+ individuals (5,624) were sequenced. We have included this estimation in the main document in the revised manuscript (line 110 in the marked document), which is an expected percentage of sequences included in phylogenetic analyses. Although, we found similar distributions of the samples sequenced from outside and inside of the geographical HIV cluster (Figures S7 and S8 in supplementary materials), we understand the reviewer's concern and included a more detailed description of the implications of incomplete sequencing of the entire samples in the limitations section of the revised manuscript (lines 391-404 in the marked document).

4. Sequencing and phylogenetic analysis – what was the definition of a cluster used? What thresholds were used?

- We have included a more detailed description of the phylogenetic methos used in the study including a definition of a phylogenetic cluster in the revised version of the manuscript (lines 108-131 in the marked document)

5. Figure 3A – not sure of the Pearson corr was the most appropriate given a number of outliers in the dataset.

- We understand the reviewer concern. We used Pearson correlation mostly for illustrative purposes and to highlight a simple positive correlation between HIV prevalence and node degree, which was later better assessed using network analysis. As we mentioned previously we choose this estimation for simplicity and illustrative

purposes but it does not have a substantial impact on the main conclusions of our results.

Reviewer #2:

This paper blends spatial epidemiology and phylogenetic methods to investigate the spread of HIV in the Hlabisa region of South Africa. They demonstrate that a small region with the highest HIV prevalence and incidence also turns out to the location of many highly connected nodes in an Hiv phylogeny for the region. The methods were novel and interesting and most of the paper as written clearly; the results were interesting ad the figures are great. Please include line numbers in any future manuscripts!

- We thank the thoughtful appraisal of our work conducted by the reviewer and the positive feedback provided. We followed the reviewer's suggestion and included line numbers in the marked version of the manuscript.

Major comments

- *Phylo/Network analysis. No description of what constitutes a cluster or how transmission links were assessed. The reader is pointed to the Sup Info but even in the Sup Info I could not find a clear explanation of either. It just says "the Phylotype approach" (with no reference). Please describe in more detail and clearly how transmission links were assessed, in the main body of the manuscript. I think you then also create a genetic network from the phylo links, but this is not explained either*

- We have included a more detailed description of the phylogenetic methos used in the study including a definition of a phylogenetic cluster in the revised version of the manuscript (lines 108-131 in the marked document).

- *How do relative epidemic sizes influence these results? E.g. prevalence is highest in your geo cluster so you just have more cases from there, therefore more people link to it*

- We thank the reviewer for the thoughtful comments. We agree with the reviewer that the high HIV prevalence in the HIV geocluster increases the likelihood of link formation from these areas, but this condition seems to be insufficient condition for the attractiveness from this area observed in the network configuration depicted of this study. For that reason we generated the microsimulation models to assess the randomness of the network configuration. These microsimulations suggested that the geocluster generated an attractiveness not explained by the HIV prevalence only. We have included a more detailed description of these microsimulations in the revised version of the manuscript (lines 187-200 in the marked document). Moreover, in the study that accompanies this manuscript that is also currently under review in this journal, using partnership formation data we found similar structure of the contact network with a significant attractiveness of the geocluster for the formation

of the links, similar to what was observed in this study. We are currently exploring potential socieoeconomic and demographic variables that could explain the attractiveness and high role of the geocluster in the network configuration.

Abstract

- *Ambitious sounding and exciting, but in the first sentence I read "HIV geographical clusters" and I am not sure what that means. I think the terms in the abstract probably need to be clearly stated (geo cluster, transmission links). In fact, although the authors are careful with their use of words throughout, they need to explicitly explain what they are calling a cluster because the term cluster already had a meaning in HIV phylogenetics.*

- We thank the reviewer for noticing this inconsistency that could generate confusion. We have now clarified the meaning of HIV geographical cluster in the abstract, and also in several parts of the manuscript.

Background

- *No references to any published spatial epi/phylo papers, e.g. https://pubmed.ncbi.nlm.nih.gov/34159215/*

- We have included relevant literature regarding phylogeographic studies in similar settings in Africa, particularly the study conducted in Rakai, Uganda:
  -Grabowski MK, Lessler J, Bazaale J, Nabukalu D, Nankinga J, Nantume B, et al. Migration, hotspots, and dispersal of HIV infection in Rakai, Uganda. Nature communications. 2020;11(1):1-12
  -Ratmann O, Kagaayi J, Hall M, Golubchick T, Kigozi G, Xi X, et al. Quantifying HIV transmission flow between high-prevalence hotspots and surrounding communities: a population-based study in Rakai, Uganda. The Lancet HIV. 2020;7(3):e173-e83.
  -Bbosa N, Ssemwanga D, Nsubuga RN, Salazar-Gonzalez JF, Salazar MG, Nanyonjo M, et al. Phylogeography of HIV-1 suggests that Ugandan fishing communities are a sink for, not a source of, virus from general populations. Scientific reports. 2019;9(1):1-8.
  -Kiwuwa-Muyingo S, Nazziwa J, Ssemwanga D, Ilmonen P, Njai H, Ndembi N, et al. HIV-1 transmission networks in high risk fishing communities on the shores of Lake Victoria in Uganda: A phylogenetic and epidemiological approach. PloS one. 2017;12(10):e0185818.

- *Typo in last line of background (or should be of)*

- We thank the reviewer for noticing this. We have corrected this typo accordingly (line 58 in the marked document)

Methods

- *P6 para 3 than it would be expected should be than would be expected*

- We thank the reviewer for noticing this. We have corrected this typo accordingly (line 146 in the marked document)

- *Add a subsection for microsimulation, even if most of that detail is in supplementary. Also include a brief explanation here of random vs gravity and what they mean because it's important to understanding the results.*

- We have followed the reviewer's recommendation and included a subsection for the microsimulations in the methods and results of the revised document (lines 187-206 and 275-283 in the marked document)

Results

- *Figure 2 legend D) should be bolded, ad a full stop at the end but great figure. Maybe clarify in legend that "HIV cluster" means "HIV geographical cluster"*

- We thank the reviewer for noticing this. We have corrected these inconsistencies accordingly

- *P 10 top – you refer to figure Sup2D, but that figure doesn't exist*

- We thank the reviewer for noticing this. We have corrected it in the revised version of the manuscript (line 174 in the marked document)

Discussion

- *First sentence problem with grammar*

- We have rephrased this sentence for grammar correctness (line 285 in the marked document)

- *P11 second paragraph, do you mean the geographic HIV cluster?*

- Yes, we have clarified this in the revised version of the document (line 293 in the marked document)

- *The discussion is too long, slightly repetitive and the sentences are too long, I suggest you try to tighten it up a bit.*

- We thank the reviewer for the suggestion. For clarity and consistency, we have included subsections in the discussion for the limitations and conclusions of the

study in the revised version of the document (lines 376-430 in the marked document)

- *The time from infection and directionality results come into the discussion but have not been discussed in the results only in the supplementary materials, and their relevance to the manuscript is unclear without reading the supplementary*

- We thank the reviewer for noticing this. We have removed several supplementary results that were not discussed in the main document and had no influence in the main results and conclusions of this study.

References

- *Ref 4 is missing*

- We thank the reviewer for noticing this. The reference has been included in the revised version of the document.

Supplementary Materials

- *Amazingly detailed and clear, I really applaud your efforts to explain everything in so much detail*

- We thank the reviewer for the positive feedback. We believe it is important to include a clear description of methods and results for replicability and interpretation of the methods used in this study

- *Don't really understand the order of the figures in relation to the main manuscript*

- We have modified the order of the figures and removed some other figures to be consistent with the content of the main manuscript.

- *Very extensive – some things don't seem to even be in the main manuscript, like directionality of links*

- As mentioned in a previous comment, several sections of the supplementary materials that had no implications in the main results of our study were removed from the updated version of this document.
