# Supplementary material for: The role of high-risk geographies in the perpetuation of the HIV epidemic in rural South Africa: A spatial molecular epidemiology study
Source: PLOS Glob Public Health. 2022 Feb 22;2(2):e0000105. doi: 10.1371/journal.pgph.0000105 (PMC10021703; doi:10.1371/journal.pgph.0000105)
Supplement: Attachment — Submitted filename: Reviewer_Comments_102421.pdf [file pgph.0000105.s003.pdf]

The role of high-risk geographies in the perpetuation of the HIV epidemic in rural South Africa: A spatial molecular epidemiology study

Diego F. Cuadros, Tulio de Oliveira, Tiago Gräf, Dennis M. Junqueira, Eduan Wilkinson, Philippe Lemey, Till Bärnighausen, Hae-Young Kim, Frank Tanser

RESPONSE TO REVIEWERS' COMMENTS

We appreciate the opportunity to review our work to the journal. Please find below a point-by-point reply that addresses each of the editor's suggestions. We have also incorporated these suggestions in the revised manuscript as noted below.

Journal Requirements:

Please review your reference list to ensure that it is complete and correct. If you have cited papers that have been retracted, please include the rationale for doing so in the manuscript text, or remove these references and replace them with relevant current references. Any changes to the reference list should be mentioned in the rebuttal letter that accompanies your revised manuscript. If you need to cite a retracted article, indicate the article's retracted status in the References list and also include a citation and full reference for the retraction notice.

- We have reviewed the reference list and confirm that it is complete and correct.

Additional Editor Comments:

I have reviewed the revised manuscript and response to the reviewer comments, and all of the major issues identified during the initial review have been addressed. I applaud the authors for a thorough and thoughtful response to the prior review. Two minor issues remain:

1. Following reporting guidelines for molecular epidemiology studies, the title should clearly state that this is a molecular epidemiology study. Please see:

Field N, Cohen T, Struelens MJ, Palm D, Cookson B, Glynn JR, et al. Strengthening the Reporting of Molecular Epidemiology for Infectious Diseases (STROME-ID): an extension of the STROBE statement. The Lancet Infectious Diseases. 2014 Apr;14(4):341–52.

- We have followed the editor's suggestion and changed the title of the manuscript to state that this was a molecular epidemiology study.

2. The last sentence in the abstract appears to be a run-on sentence, and it contains assertions about the effect of an intervention that is not directly part of this study. Given that

study is observational and descriptive in nature, please rephrase this sentence to be more nuanced about the impact of a hypothetical intervention. The last sentence of the Conclusion (lines 427 - 430) should likewise be revised, since the intervention mentioned in that sentence was not tested in this study.

- We have followed the editor's suggestion and removed the last sentence of the abstract (lines 12-14 in the marked document). We have also toned down the last sentence of the conclusions as suggested by the viewer (lines 410-412 in the marked document).
